# Comparative surgical risk between type of trampoline (size and place) and type of patients (age and sex) in trampoline related injury: a systematic review and indirect meta-analysis

**DOI:** 10.1186/s13102-020-00185-w

**Published:** 2020-07-06

**Authors:** Janisa Andrea Muljadi, Kornkit Chaijenkij, Alisara Arirachakaran, Jatupon Kongtharvonskul

**Affiliations:** 1 Mater Dei School, Bangkok, Thailand; 2 Orthopedic department, College of Sports Science and Technology, Mahidol University, Bangkok, Thailand; 3 Orthopedics Department, Bumrungrad Hospital, Bangkok, Thailand; 4 Section for Clinical Epidemiology and Biostatistics, Faculty of Medicine Ramathibodi Hospital and Orthopedic department, Payathai3 Hospital, Bangkok, Thailand

**Keywords:** Trampoline, Trampoline related injury, Full or mini trampoline, Park or home, Surgery, Systematic review, Meta-analysis

## Abstract

**Background:**

Despite its high risk of injury, many people are still favor trampolining. However, currently there is no consensus as to which type of trampoline and which type of participant is more likely to have a trampoline related injury that will require surgical management.

**Methods:**

This systematic review and meta-analysis aims to assess and compare the factors that cause trampoline injuries requiring surgical treatment. These include the place of the trampoline (park versus home), size of the trampoline (full versus mini), the age of the participant (child versus adult) and the sex of the participant (male versus female). The clinical outcomes measured are surgical management after trampoline injury. This systematic review was conducted according to the PRISMA guidelines.

**Results:**

Relevant studies that reported surgery after trampoline injury of either group were identified from Medline and Scopus from inception to May 14, 2019. Sixteen studies were included for the analysis of surgery after trampoline injury; a total of 4491 and 1121 patients were treated conservatively and surgically. The total surgery rate per patient was 31% (95% CI: 16, 46%) in all patients. The surgery rate was 0.3 (95% CI: 0.03, 0.58) and 0.06 (95% CI: 0.04, 0.09) in the full and mini size trampoline groups. There were 0.36 (95% CI: 0.06, 0.67) and 0.11 (95% CI: 0.0, 0.22) in the park and home trampoline groups. The surgery rates were 0.33 (95% CI: 0.14, 0.53), 0.24 (95% CI: 0.07, 0.11), 0.49 (95% CI: 0.47, 0.51) and 0.38 (95% CI: 0.22, 0.53) in children, adults, females and males respectively. Indirect meta-analysis shows that full size trampolines provided a 6.0 times higher risk of surgery (95% CI: 3.7, 9.7) when compared to mini size trampolines. Park trampolines had a higher risk of surgery of 2.17 (95% CI: 1.70, 2.78) when compared to home trampolines. In terms of age and sex of participants, there value was significantly higher at 1.65 (95% CI: 1.35, 2.01) and 1.54 (95% CI: 1.36, 1.74) in children compared to adults and females compared to males. From all the statistical data we summarized that the full size trampoline injuries have a 6 times higher risk of requiring surgery when compared to mini size trampoline injuries. Park trampoline use carries a 2 times higher risk of requiring surgery when compared to home trampoline use. In terms of age and sex of the participant, there is a 1.5 times significantly higher risk of injury in children compared to adults, and females when compared to males.

**Conclusion:**

In trampoline related injuries, full size, park trampoline, children and females had higher surgery rates when compared to mini size, home trampoline, adult and male majority in indirect meta-analysis methods.

## Background

The first trampoline related injuries were reported in 1956 by Zimmerman [1] and in 1960 by Ellis et al. [2]. Spinal cord injuries are among the most severe injury associated with trampolines. Most of these injuries involve the cervical spine and result in quadriplegia [3–5]. The growing popularity of trampolines has caused significant increases in the number of injuries associated with their use [6]. During the period of 2000–2005, trampoline injury cases went up sharply at the rate of 113% compared to the past 5 years average [7–9]. Trampolines are very popular among children. The majority of trampoline injury patients are children, whereas the adult patients are less than 1 to 25%. Most of the injuries occurred on full-sized trampolines, but home trampolines are should be commonly involved. The causes of trampoline injuries that frequently happen are collision with another person on trampoline, awkward landing and falling off from the trampoline to the ground surface or building structure. Only two previous studies [10, 11] explored the epidemiology risk factors associated with trampoline related injuries. First study [10] compared park with domestic trampoline injuries and the results was reported that jump parks trampoline-related injury had higher risk of fractures or dislocations and surgical interventions when compared to home trampolines. Another study [11] has reported results of mini-trampolines compared with full-sized trampolines, children compared with adults. The result shows that the use of full-sized trampolines had lower risk of injury than mini-size and young children had higher risk of injury than older. However, both studies have a small number of patients that may not be representative of trampoline-related injuries and severity of patients was determined by admission rate which is not appropriate [10, 11]. Moreover there still no information about other epidemiology risk factor associated of trampoline related injuries. Therefore, this systematic review and meta-analysis aims to assess and compare risk of surgery related after trampoline injury between place of trampoline (park versus home), size of trampoline (full versus mini), age of participant (children versus adult) and sex of participant (male versus female majority). This information may lead to increased public awareness of the potential for serious injuries and permanently disabling outcomes for those who participate in recreational trampoline use.

## Methods

Medline and Scopus databases were used to identify relevant studies published in English since the date of inception to May 14, 2019. The PubMed and Scopus search engines were used to locate studies with the following search terms: Trampoline related injury. References from the reference lists of included trials and previous systematic reviews were also explored. The review protocol has been registered at the international prospective register of systematic review (PROSPERO ID: 147234).

### Inclusion criteria

Clinical studies (e.g., observational, cross-sectional, cohort or randomized controlled trial (RCT)) that reported the type of treatment, whether conservative or surgical, after trampoline-related injury were eligible if they met the following criteria:

- Reported treatment conservatively or surgically after trampoline-related injury.
- Had sufficient data to extract and pool, i.e. the reported mean, standard deviation (SD), the number of subjects according to treatments for continuous outcomes, and the number of patients according to treatment for dichotomous outcomes.

### Exclusion criteria

- The reference lists of the retrieved articles were also reviewed to identify publications on the same topic. Where there were multiple publications from the same study group on the same population, the most complete and recent results were used.
- Non-English studies were excluded.

### Data extraction

Two reviewers (J.M. and J.K.) independently performed data extraction using standardized data extraction forms. General characteristics of the study (i.e. mean age, gender, body mass, location of injury, size of trampolines, mechanism of injury (fall, collision, fell off, contact with structure, unknown), type of injury (sprain, fracture, dislocation, concussion, other), region of injury (spine, upper extremity, head, trunk, lower extremity, other), and length of hospital stay were extracted. All dichotomous outcomes (any type of surgery) were also extracted. Any disagreements were resolved by discussion and consensus with a third party (A.A.).

### Outcomes of interest

The outcomes of interest included surgery or conservative treatment after trampoline injury. These outcomes were measured as reported in the original studies which were surgical (Fixation of extremity fracture, spine surgery, head and neck surgery, thoracic and abdominal surgery) and conservative (medication, casting, splint, observation) treatment which included patients who were outpatients and inpatients.

### Statistical analysis

For dichotomous outcomes (surgery), the prevalence was pooled and calculated using the Mantel-Haenszel analysis method. Heterogeneity of mean differences was checked using the Q statistic and the degree of heterogeneity was also quantified using the I_2_ statistic [12]. If heterogeneity was significant or I_2_ > 25%, the pooled prevalence was estimated using a random effects model, otherwise a fixed effects model was applied. Meta-regression analysis was then applied to explore causes of heterogeneity [12, 13]. Coverable parameters i.e. mean age, gender, body mass, location of injury, size of trampolines, mechanism of injury (fall, collision, fell off, contact with structure, unknown), type of injury (sprain, fracture, dislocation, concussion, other), region of injury (spine, upper extremity, head, trunk, lower extremity, other), and length of hospital stay were considered in the meta-regression model. Power of the test for meta-regression was also assessed [14]. The odds ratio (OR) were estimated by indirect meta-analysis using a random effects model, If heterogeneity was significant or I2 > 25%, otherwise a fixed effects model was applied otherwise a fixed effects model was applied. All analyses were performed using STATA version 15.0 [15]. A *p*-value < 0.05 was considered statistically significant, except for the test of heterogeneity where < 0.10 was used.

## Results

**Fig. 1:**
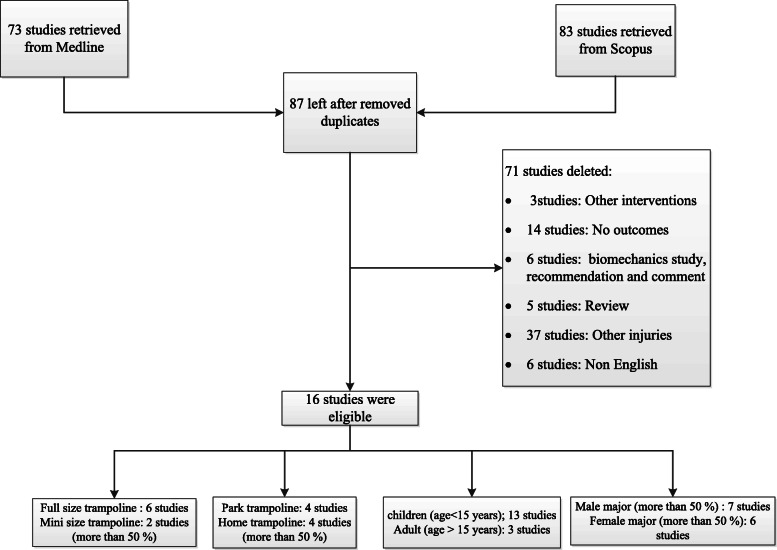
Flow of the study

Seventy three and 83 studies were identified from Medline and Scopus respectively, as described in Fig. 1. Sixty-nine studies were duplicates, leaving 87 studies for review of titles and abstracts. Of these, 16 articles [10, 11, 16–29] were relevant and the full papers were retrieved. Characteristics of these studies are described in Table 1. Seventy-one studies were deleted under exclusion criteria; 3, 14, 6, 5, 37 and 6 studies were other intervention, no outcomes, biomechanics, review, other injuries and no English language, respectively. Sixteen studies were included for the analysis of trampoline-related injury; 14 studies [16–29] were retrospective cohort and 2 studies [10, 11] were comparative cohort studies. All 14 studies reported conservative and surgical management. Four studies, 2, 2, 2, 1, 1, 1, 1, 1 and 1 study were reported from America, Australia, Ireland, Korea, Denmark, Hong Kong, Canada, Austria, Finland and United Kingdom, respectively. Six and two studies were included for the analysis of full and mini size trampolines. Four studies each were included for analysis of park versus home trampolines. Thirteen studies included mostly children, and another three studies included mostly adults. Seven studies were mostly male, while 6 studies were mostly female. The lower extremities were the most common sites of injury (42%) whereas the spine was the least common site of injury (4%). The most common mechanism of injury was falling on the trampoline (30%). Fractures were the most common injury (40%) while dislocation was the least common injury (4%). Mean age and percentages of male patients varied from 5.25 to 25 years and 37 to 71% (Table 1).

**Table 1:** Characteristics of included studies

Author	Years	Nationality	Patients (N)	Age (Years)	Male (%)	Trampoline size		Place			Mechanism of injury					Injury region				
						Full (%)	Mini (%)	Park (%)	Home (%)	Other (%)	Fall	Collision	Fell of tram	Contact with structure	Other	Head	Spine	Upper extremities	Trunk	Lower extremities
Doty J	2019	America	150	20.5	78	–	–	1	–	–	111	7	12	20	–	–	3	16	–	64
Doty Y	2019	America	289	21.5	157	–	–	–	1	–	171	43	46	29	–	–	62	97	–	130
Cho MJ	2019	Korea	178	–	–	–	–	–	–	–	41	32	10	13	92	26		34	8	111
Choi ES	2018	Korea	2799	5.25	1526	–	–	0.76	0.17	0.07	754	542	364	356	783	679	55	678	68	1318
Thi Huynh AN	2018	Denmark	113	7.5	–	–	–	–	–	–	–	–	–	–	–	3	18	56	1	43
Yule MS	2016	Hong Kong	344	7	–	–	–	–	–	–	–	–	–	–	–	2		239	41	64
Arora V	2016	Australia	50	25	35	–	–	–	–	–	–	–	–	–	–		20	1	–	35
Sandler G	2011	Australia	383	7	193	–	–	–	0.90	0.1	294	32	–	–	57	81	5	25	–	41
Leonard H	2009	Canada	7	11.6	5	–	–	–	–	–	2	–	–	4	1	–	7	–	–	–
Eberl R,	2009	Austria	265	7.85	122	0.38	0.62	–	–	–	102	56	36	32	39	24	–	76	29	136
Rattya J	2008	Finland	76	7.65	35	0.97	0.01	–	0.88	0.12	25	8	25	5	3	–	5	45		28
Hurson C	2007	Ireland	101	8.45	41	0.98	0.02	0.05	0.95	–	23	13	31	9	15	8	–	38	4	36
Mcdermott C	2006	Ireland	88	8.75	33	1	–	0.57	0.38	0.05	34	34	13	6	1	–	–	61		27
Shankar A	2006	UK	168	9.8	64	–	–	–	–	–	–		–	–	–	26	14	71	42	41
Shields BJ	2005	America	137	13.9	51	–	1	0.30	0.58	0.12	64	23	23	27	–	34	4	25	14	60
Shields BJ	2005	America	143	11	70	1	–	0.03	0.89	0.08	65	27	4	47	–	20	12	36	15	60
Larson BJ	1995	America	217	16.6	109	1	–	–	–	–	123	–	63	–	31	46	–	79	19	73
Woodward GA	1992	America	114	8.25	63	0.98	0.02	0.57	0.42	0.01	43	13	33	6	19	28	16	36	–	27

### Pooled prevalence of treatment (conservative and surgery) related trampoline injury

Overall, there were 5622 patients (4233 in the conservative group and 1379 in the surgery group). The total surgical rate per patient was 0.69% (95% CI: 0.54, 0.84%) and 0.31% (95% CI: 0.16, 0.46%) in all patients (Table 2).

**Table 2:** Estimation of the pooled prevalence of treatment (conservative and surgery) related trampoline injury

Author	Year	Hospital stay	N	Treatment	
				Conservative	Surgery
Doty J	2019	6.5 (7.1)	150	131	19
Doty Y	2019	1.25 (0.3)	289	269	20
Cho MJ	2019	–	178	166	12
Choi ES	2018	–	2799	2537	262
Thi Huynh AN	2018	–	113	100	13
Yule MS	2016	–	344	183	151
Arora V	2016	7.3 (5.8)	50	12	38
Sandler G	2011	10.2 (10.8)	383	147	236
Leonard H	2009	8.1 (9.0)	7	3	4
Eberl R,	2009	–	265	248	17
Rattya J	2008	5.5 (5.8)	76	45	31
Hurson C	2007	6.1 (5.1)	101	89	12
Mcdermott C	2006	2.0 (0.9)	88	52	36
Shankar A	2006	–	168	6	162
Shields BJ	2005	–	137	137	0
Shields BJ	2005	–	143	143	0
Larson BJ	1995	–	217	204	13
Woodward GA	1992	–	114	19	95
**Pooled prevalence of treatment related trampoline injury (95%CI)**				**0.69 (0.54, 0.84)**	**0.31 (95%CI: 0.16, 0.46)**

### Full versus mini size trampoline

**Fig. 2:**
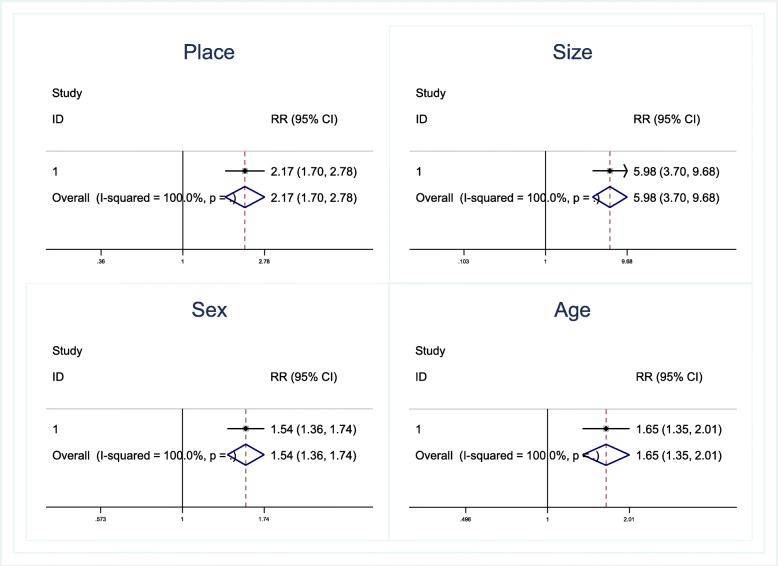
Comparison of prevalence of trampoline related surgery between place of trampoline, size of trampoline, sex and age of injury participants

There were a total of 739 and 402 patients in full and mini size trampoline studies. There were 187 patients in the full size group and 17 patients in the mini size group that had undergone surgery for trampoline injuries. The surgery rates per patient with injuries from full versus mini size trampolines were 0.3% (95% CI: 0.03, 0.58%) and 0.06% (95% CI: 0.04, 0.09) (Table 3). By indirect meta-analysis, the full sized trampoline had a higher risk of requiring surgery by 6.0 (95% CI: 3.7, 9.7) when compared to the mini sized trampoline (Fig. 2 and Table 4).

**Table 3:** Estimation of subgroup analysis of the pooled prevalence of treatment (conservative and surgery) related trampoline injury

Author	Year	N	Treatment	
			Conservative	Surgery
Rattya J	2008	76	45	31
Hurson C	2007	101	89	12
Mcdermott C	2006	88	52	36
Shields BJ,	2005	143	143	0
Larson BJ	1995	217	204	13
**Pooled prevalence of treatment related full size trampoline injury (95% CI)**			**0.70 (95% CI: 0.42, 0.97)**	**0.3 (95% CI: 0.03, 0.58)**
Eberl R,	2009	265	248	17
Shields BJ	2005	137	137	0
**Pooled prevalence of treatment related mini size trampoline injury (95% CI)**			**0.94 (95% CI: 0.91, 0.97)**	**0.06 (95% CI:0.04, 0.09)**
Doty J	2019	150	131	19
Choi ES	2018	2799	2537	262
Mcdermott C	2006	88	52	36
Woodward GA	1992	114	19	95
**Pooled prevalence of treatment related park trampoline injury (95% CI)**			**0.64 (95% CI: 0.33, 0.94)**	**0.36 (95% CI: 0.06, 0.67)**
Doty J	2019	289	269	20
Rattya J	2008	76	45	31
Hurson C	2007	101	89	12
Shields BJ	2005	137	137	0
Shields BJ	2005	143	143	0
**Pooled prevalence of treatment related home trampoline injury (95% CI)**			**0.89 (95% CI: 0.78, 1.00)**	**0.11 (95% CI: 0.0, 0.22)**
Cho MJ	2019	178	166	12
Choi ES	2018	2799	2537	262
Thi Huynh AN	2018	113	100	13
Yule MS	2016	344	183	151
Sandler G	2011	383	147	236
Leonard H	2009	7	3	4
Eberl R	2009	265	248	17
Rattya J	2008	76	45	31
Hurson C	2007	101	89	12
Mcdermott C	2006	88	52	36
Shankar A	2006	168	6	162
Shields BJ	2005	137	137	0
Shields BJ	2005	143	143	0
Woodward GA	1992	114	19	95
**Pooled prevalence of treatment of children related trampoline injury (95% CI)**			**0.67 (95% CI: 0.47, 0.86)**	**0.33 (95% CI: 0.14, 0.53)**
Doty J	2019	150	131	19
Doty J	2019	289	269	20
Arora V	2016	50	12	38
Larson BJ	1995	217	204	13
**Pooled prevalence of treatment of adult related trampoline injury (95% CI)**			**0.76 (95% CI: 0.62, 0.91)**	**0.24 (95% CI: 0.07, 0.11)**
Eberl R,	2009	265	248	17
Rattya J	2008	76	45	31
Hurson C	2007	101	89	12
Mcdermott C	2006	88	52	36
Shankar A	2006	168	6	162
Shields BJ	2005	137	137	0
Shields BJ	2005	143	143	0
**Pooled prevalence of treatment of female related trampoline injury (95% CI)**			**0.51 (95% CI: 0.49, 0.53)**	**0.49 (95% CI: 0.47, 0.51)**
Doty J	2019	150	131	19
Doty J	2019	289	269	20
Choi ES	2018	2799	2537	262
Arora V	2016	50	12	38
Sandler G	2011	383	147	236
Leonard H	2009	7	3	4
Larson BJ	1995	217	204	13
Woodward GA	1992	114	19	95
**Pooled prevalence of treatment of female related trampoline injury (95% CI)**			**0.62 (95% CI: 0.47, 0.78)**	**0.38 (95% CI: 0.22, 0.53)**

**Table 4:** Comparisons of prevalence of surgery in trampoline related injury between full and mini size, park and home, children and adult, female and male majority

Author	Surgical risk				OR	95% CI
	Full size		Mini size			
	Conservative	Surgery	Conservative	Surgery		
Pooled study	552	187	385	17	6.0	3.7, 9.7
	**Park**		**Home**			
	conservative	surgery	conservative	surgery		
Pooled study	2886	648	683	63	2.17	1.70, 2.78
	**Children**		**adult**			
	conservative	surgery	conservative	surgery		
Pooled study	3875	1031	616	90	1.65	1.35, 2.01
	**Female majority**		**Male majority**			
	conservative	surgery	conservative	surgery		
Pooled study	720	258	3322	687	1.54	1.36, 1.74

#### Park versus home trampoline

There were a total of 3534 and 746 patients in park and home trampoline studies, with 648 patients in the park group and 63 patients in the home group who had undergone surgery for trampoline injury. The surgery rate per patient of park and home trampolines were 0.36% (95% CI: 0.06, 0.67%) and 0.11% (95% CI: 0.0, 0.22) (Table 3). By indirect meta-analysis, park trampolines had a higher risk of requiring surgery by 2.17 (95% CI: 1.70, 2.78) when compared to home trampolines (Fig. 2 and Table 4).

### Age and sex associated surgery

There were a total of 4916 patients in the age group lower than 15 years of age (children) and 706 patients in the age group more than 15 years of age (adults), with 978 patients in the female group and 4009 patients in the male group. One thousand thirty-one patients in the children group and 90 patients in the adult group had undergone surgery in the trampoline injury patients. For sex, 258 patients in the male group and 687 patients in the female group had undergone surgery for trampoline-related injury.

The surgery rate per patient of for children, adult, female and male groups were 0.33% (95% CI: 0.14, 0.53%), 0.24% (95% CI: 0.07, 0.11), 0.49 (95% CI: 0.47, 0.51) and 0.38 (95% CI: 0.22, 0.53) (Table 3). By indirect meta-analysis, children and females had a higher risk of requiring surgery by 1.65 (95% CI: 1.35, 2.01) and 1.54 (95% CI: 1.36, 1.74) when compared to adult and males (Fig. 2 and Table 4).

#### Sources of heterogeneity

Meta-regression was applied for exploring the cause of heterogeneity by fitting a co-variable (i.e., age, percentage of female patients, mechanism of injury, site of injury, size of trampoline and place of injury), and meta-regression was applied to assess this. None of the co-variables could explain the heterogeneity.

## Discussion

From the current available evidence, this systematic review and meta-analysis has shown the following: full size trampoline injuries have a 6 times higher risk of requiring surgery when compared to mini size trampoline injuries. Park trampoline use carries a 2 times higher risk of requiring surgery when compared to home trampoline use. In terms of age and sex of the participant, there is a 1.5 times significantly higher risk of injury in children compared to adults, and females when compared to males.

From previous published studies [9, 11, 23, 25, 26, 30, 31], the American Academy of Pediatrics (AAP) issued a policy statement in 1977 recommending “that trampolines be banned from use as part of the physical education programs in grammar schools, high schools, and colleges, and also be abolished as a competitive sport.” [31] Only three studies have reported risk factors associated with trampoline injury, with the first study reported in 2005 by Shield et al. [11], in which they reported the injury patterns were similar for mini and full sized trampolines, although mini trampoline-related injuries were less likely to require admission to the hospital. Whereas this current study has a significantly lower amount of injuries in mini-trampoline when compared to full size due to the sufficient sample size to correct the type 2 error and use conservative or surgical management to separate patients in two groups by severity of their injury (low and high severity injury) instead of admission. The second study is by Choi et al., which reported a higher number of pediatric trampoline injuries and trampoline park injuries, while ages at injury have tended to be lower, which are results that correspond with this study. Today, the widespread use of trampolines has led to a significant increase of related trauma. There, we suggest modify or additional recommendation in the policies to prevent trampoline injuries according to the results of previous published studies and this meta-analysis. Firstly, all full size and mini trampolines use should follow the policy recommendations of the American Academy of Pediatrics (AAP). Use of the mini trampoline could lower the risk of injury when compared to using full sized trampolines. Secondly, children who are aged below 15 years should be under adult supervision and always wear protection (e.g., knee pad, wrist pad and elbow pad protector) of the lower and upper extremities to prevent fracture or dislocation [9], which is the most common cause of injury requiring surgery in this study. Thirdly, jump park trampolines should be banned to lower the risk of injury then we recommend use of home trampolines. Lastly, muscle strength and proprioceptive sensation training should be done before and after jumping on the trampoline in all children to prevent injury, especially in the female sex. We want this study to be the turning point in changing the policy maker belief then the newest AAP recommendation should include the result of this systematic review in the future.

The strength of this study is that adequate methodology was used for systematic reviews in accordance with PRISMA guidelines [32] as well as providing exploration and reduction of the heterogeneity of the studies using subgroup analysis and adequate statistical analysis.

Moreover, this study has conclusive evidence about risk factors such as size, place, age and sex that should be selected to decrease risk of surgery after trampoline-related injury. Some limitations in this study are that the number of patients & studies was also not high. Another limitation is this study did not pool an important outcome such as frequency of using trampoline and injury rates with multiple users on the trampoline at the same time due to the fact that there was insufficient data. Further research that assesses a larger sample size of RCTs should be done to see any significance of complications.

## Conclusions

In trampoline-related injury, full size and park trampolines, children and females had higher surgery rates when compared to mini size and home trampolines, adults and males with indirect meta-analysis methods. This result recommended use of the mini trampoline could lower the risk of injury, children should be under adult supervision and always wear protection to prevent lower and upper extremities injury, Jump park trampolines should be banned and only of home trampolines should be used. Prospective randomized controlled studies are needed to confirm these findings as the current literature is still insufficient.

## Data Availability

All data generated or analyzed during this study are included in this published article.

## Abbreviations

PRISMA: Preferred Reporting Items for Systematic Reviews and Meta-Analyses
RCT: randomized controlled trial
SD: standard deviation
OR: odds ratio
AAP: American Academy of Pediatrics
